# Can Optimum Solar Radiation Exposure or Supplemented Vitamin D Intake Reduce the Severity of COVID-19 Symptoms?

**DOI:** 10.3390/ijerph18020740

**Published:** 2021-01-16

**Authors:** Joji Abraham, Kim Dowling, Singarayer Florentine

**Affiliations:** 1 School of Engineering, Information Technology and Physical Sciences, Mount Helen Campus, Federation University Australia, Ballarat, VIC 3353, Australia; k.dowling@federation.edu.au; 2 Department of Geology, University of Johannesburg, Johannesburg 2006, South Africa; 3 School of Science, Psychology, and Sport, Centre for Environmental Management, Mount Helen Campus, Federation University Australia, Ballarat, VIC 3353, Australia; s.florentine@federation.edu.au

**Keywords:** acute respiratory distress syndrome (ARDS), acute respiratory tract infection (ARTI), calcifediol, calcitriol, coronavirus, environment, human health, infectious disease, pandemic, SARS-CoV-2

## Abstract

The foremost mortality-causing symptom associated with COVID-19 is acute respiratory distress syndrome (ARDS). A significant correlation has been identified between the deficiency in vitamin D and the risk of developing ARDS. It has been suggested that if we can reduce or modify ARDS in COVID-19 patients, we may significantly reduce the severity of COVID-19 symptoms and associated mortality rates. The increased mortality of dark-skinned people, who have a reduced UV absorption capacity, may be consistent with diminished vitamin D status. The factors associated with COVID-19 mortality, such as old age, ethnicity, obesity, hypertension, cardiovascular diseases, and diabetes, are all found to be linked with vitamin D deficiency. Based on this review and as a precautionary measure, it is suggested that the adoption of appropriate and safe solar exposure and vitamin D enriched foods and supplements should be considered to reduce the possible severity of COVID-19 symptoms. Safe sun exposure is deemed beneficial globally, specifically in low and middle-income countries, as there is no cost involved. It is also noted that improved solar exposure and vitamin D levels can reduce the impact of other diseases as well, thus assisting in maintaining general human well-being.

## 1. Introduction

In December 2019, a novel coronavirus (nCoV) strain [1], initially identified as ‘severe acute respiratory syndrome coronavirus’ (SARS-CoV), and later named as SARS-CoV-2, became the focus of attention all around the world with the resulting disease named ‘Coronavirus Disease 2019 (COVID-19)’ by the World Health Organisation (WHO) [2]. Despite the continued efforts from governments across the world to slow down the rates of infection and fatality, the disease has become a pandemic. As of the 26 December 2020, more than 80 million confirmed cases and more than 1.8 million fatalities across the world have been reported, with majority of the reported fatalities being in the United States (330,254), Brazil (190,488), and India (147,092) [3]. This infection rate sharply contrasts with two previously reported coronavirus exposure events in the last two decades [4].

The disease is potentially lethal to adults over 60 years of age and is particularly so for those with comorbidities such as diabetes, hypertension, heart disease, cancer, and obesity [5]. Even though vaccinations have begun in several countries, it is unclear when the vaccine will be available more widely, specifically to low-income countries. The most optimistic current estimates are mid to late 2021, and not much is known concerning how long the vaccine can protect the individual. This lag of availability and uncertainty in protection highlight the significance of finding strategies to confront and mitigate the spread and severity of COVID-19 at least for several more months. A detailed analysis of the data regarding the clinical problems of COVID-19 across the world has emphasised that immunity is a significant factor in controlling viral pathogenicity [6]. The mode of COVID-19 spreading, together with the number of fatalities across the world, has alerted the researchers to the possibility that location, climate, exposure to solar radiation, and vitamin D have a link in viral pathogenicity and mortality [7,8]. Moreover, previous studies conducted across the world have observed a significant association between vitamin D and immunity, especially in terms of acute respiratory tract infections (ARTI) and acute respiratory distress syndrome (ARDS), resulting multiple health outcomes and mortality [9,10,11].

Vitamin D is recognised as a secosteroid and is synthesised endogenously by the effect of ultraviolet B (UVB) radiation on the skin. It has well understood substantial immunomodulatory and anti-inflammatory actions in the human body [12]. Several studies have emphasized the implications of vitamin D deficiency as a contributing factor in many diseases such as diabetes mellitus [13], cardiovascular disease [14], autoimmune liver disease [8], and obesity [15]. Of importance here is that studies have shown that lung inflammation that leads to ARDS is considered to be the main cause of the deaths among COVID-19 patients, and it is the renin–angiotensin system which is very significant in making such an inflammatory response [16]. With direct relevance to this paper, it has been unequivocally observed that vitamin D substantially influences the renin–angiotensin system and leads to a reduction in the inflammatory response [17,18,19]. In a mouse model study, Kong et al. [17] identified that vitamin D could have significant influence in reducing lipopolysaccharide-induced lung injury by blocking the effects of Ang-2-Tie-2 and renin–angiotensin pathways. In support of this observation, Zhang et al. [18] and Xu et al. [19] reported that vitamin D acts as a protective substrate in alleviating the lipopolysaccharide-induced acute lung injury (ALI) by the inhibition of pro-inflammatory cytokine interleukin-6 (IL-6). Both these studies are of significance to the welfare of COVID-19 patients. In parallel with these studies, a recent meta-analysis of eight observational studies incorporating the details of 21,000 subjects revealed that there was the potentiality of increased risk of acquiring pneumonia from community sources among those with low vitamin D (<20 ng/mL) levels [20].

People with common immunodeficiency and adults detected with vitamin D deficiency have been found to be vulnerable to COVID-19 [21,22]. More than 70% of the deaths recorded in Chicago (USA) are among the African-American population, who are normally vitamin D deficient communities, specifically during the winter, probably due to skin pigmentation blocking the UV radiation [23,24]. This finding also supports similar data from the United Kingdom [25], where according to the 2011 census, only around 14% of the population in England and Wales are black and minority ethnic, but a third of the confirmed COVID-19 cases admitted to the critical care in the hospitals belong to these black minorities [25,26].

Many of the first phase worst-hit coronavirus areas are located in the same latitudinal temperature region known to have reported vitamin D deficiency. This emphasises the significance of the lack of serum vitamin D in COVID-19 fatalities [27]. Even though data from large randomised placebo-controlled double-blind, multicentre studies are lacking, the available data from observational and interventional studies give strong supportive results to the hypothesised link between vitamin D deficiency and increased infection rates of COVID-19. Apart from this observation, vitamin D treatment given to a small cohort of COVID-19 patients in Spain has revealed promising results [28].

It is already well known that vitamin D has a role in inhibiting a range of infections and inflammations, including acute respiratory tract infection (ARTI) and acute respiratory distress syndrome (ARDS). Whilst there are alternative methods of obtaining vitamin D, acquiring increased levels through solar radiation is considered most convenient and beneficial as it is completely free and natural. Unfortunately, in times of lockdown and social distancing, those who are most at risk, the elderly, and health compromised individuals face practical hurdles in obtaining vitamin D via natural means. The objective of this article is to examine published evidence showing the influence of the increase in vitamin D levels in relieving symptoms similar to COVID-19, especially ARDS and ARTI. The study has also reviewed recently published observational studies, including small pilot studies (in 2020), which have shown the association between vitamin D deficiency and COVID-19 positivity or severity. The work concludes with suggestions of various methods to improve vitamin D sufficiency among the general public to prevent severe COVID-19 symptoms and mortality.

## 2. Methodology

Several searches have been conducted in the existing literature using Google Scholar, PubMed, Scopus, Web of Science, and the preprint database Medrevix to obtain the most up to date research information regarding vitamin D and human health. The searches specifically focus on COVID-19 symptoms such as ARTI and ARDS identifying publications from the last two decades, using the terminologies (i) vitamin D and human health, (ii) vitamin D and COVID-19, (iii) vitamin D and ARTI, and (iv) vitamin D and ARDS. Most of the studies highlighting the relation between vitamin D and ARDS/ARTI were incorporated, whilst other, only generally related studies were eliminated from the review. These studies and their influence on ARDS and ARTI have been discussed, and a summary is prepared as a Table. Recent studies showing a correlation between vitamin D serum level deficiency and COVID-19 severity conducted in 2020 were also reviewed, discussed, and prepared as a separate Table. This review includes the following sections: (i) symptomatic background of COVID-19; (ii) some background information to vitamin D studies; (iii) influence of vitamin D in reducing ARTI and ARDS; (iv) vitamin D geographical context, ethnicity and age; (v) vitamin D and COVID-19; (vi) the role of vitamin D in infection control; and finally (vii) how to acquire a sufficient level of vitamin D.

## 3. The Symptomatic Background of COVID-19

Coronaviruses are a large family of single-stranded, enveloped RNA viruses generally seen in several animal species, with SARS-CoV-2 being the specific pathogen related to the current pandemic. Symptoms associated with COVID-19 usually appear after an incubation period of two to twelve days, with the majority of people showing signs within six days [4]. Apart from fever, fatigue, and myalgia, ARDS is a critical factor in 6% to 16% of COVID-19 patients and is considered the major cause of organ failure and death [29,30]. It is characterized by the critical progression of respiratory symptoms, signs including bilateral diffuse infiltrates on chest imaging, and severe hypoxemia caused by heterogeneous aetiologies [30]. There are many known causes of ARDS development, but COVID-19 patients suffer from pathogen-caused lung injury [29]. Even though medical science has progressed, the exact mechanisms behind ALI and ARDS have not yet been fully revealed, and effective pharmacological interventions are therefore not able to be developed [19]. Once ARDS occurs, effective management is difficult. As a consequence, any early intervention that can prevent or reduce either viral replication, ARTI, or ARDS will be worthwhile, and thus vitamin D has a significant role in this. In this respect, the association between vitamin D and clinical symptoms similar to COVID-19 has been mentioned in many studies (Table 1).

## 4. Some Background to Vitamin D Studies

**Figure 1:**
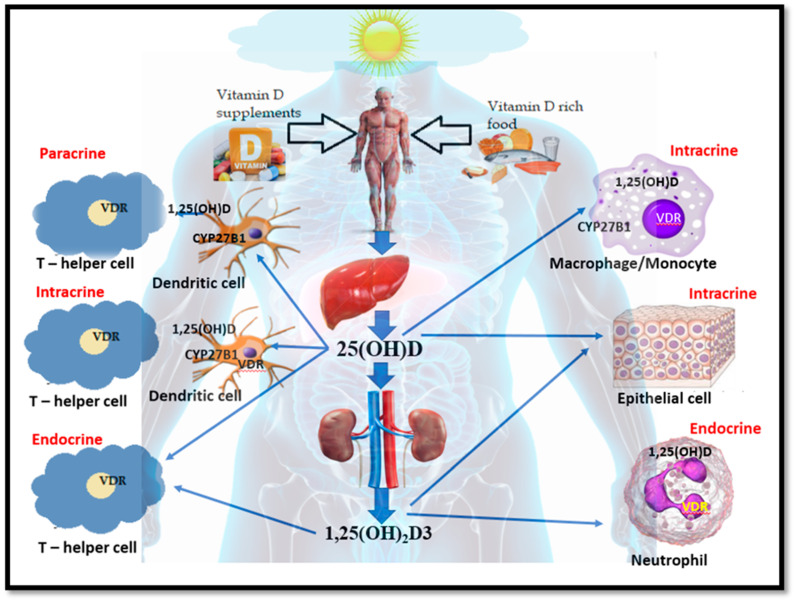
Formation of two main forms of vitamin D: the main circulating form, 25 hydroxyvitamin D (25(OH)D), and the main active form, 1ἁ,25 dihydroxy vitamin D (1,25(OH)_2_, D) as well as their interaction with the innate cells. (VDR-vitamin D receptor) (adapted from [48]).

Vitamin D3 is naturally produced as a result of the action of sunlight (UVB radiation 290–315 nm) on 7-dehydrocholesterol within the skin [39]. The material produced from this source, in addition to that absorbed from food, is inert and requires hydroxylation to circulate in the body. This reaction occurs in the liver, where it forms calcifediol (25-hydroxyvitamin D) using the enzyme D-25 hydroxylase [40]. Subsequently, this moves to the kidney for further hydroxylation, forming 1ἁ,25 dihydroxy vitamin D (1,25(OH)_2_D or calcitriol-C_27_H_44_O_3_) using the enzyme 25(OH)D-1 hydroxylase (a mitochondrial oxygenase), and this calcitriol is the major active form of vitamin D [40] (Figure 1). Vitamin D has several biological functions in the body, including (i) calcium homeostasis; (ii) the regulation of up to 1000 genes; (iii) inhibition of cellular proliferation, angiotensins, and renin production; and (iv) inducing insulin and macrophage cathelicidin and defensins productions (both have immune-modulatory effects) [41,42,43]. Studies have shown that cathelicidin has anti-microbial properties against several bacteria and viruses (including enveloped viruses) [44,45]. A mouse model study demonstrated that LL-37 reduced influenza-A virus replication [46], and a pig model study underlined that vitamin D could reduce the replication of the rotavirus in vitro and in vivo [47]. Similar studies, including data, and reports have revealed that vitamin D has a substantial role in inducing innate immunity by maintaining localised the production of antibacterial CAP (cathelicidin antimicrobial peptide) [47].

Many of the immune cells are equipped with vitamin D receptors (VDRs) [49,50]. Antigen-presenting cells such as macrophages and dendritic cells could be influenced by vitamin D (25 hydroxyvitamin D) as they can synthesize 1,25-dihydroxy vitamin D from 25-hydroxyvitamin D via 1ἁ hydroxylase (CYP27B1) [51]. Many respiratory pathogens, including SARS-CoV-2, invade the epithelial cells in the lung alveoli, making the epithelial cells as first responders to recruit neutrophils and T cells to the infection site through the activation of macrophages and dendritic cells [41]. In the absence or deficiency of vitamin D (25-hydroxyvitamin D), the first triggering mechanism would be considered to be impaired. It is hypothesised that this triggering mechanism is like a circuit breaker. In the absence of 25 hydroxyvitamin D, the circuit cannot be completed, or at best, it will be weak due to unsatisfied VDR, which may affect the innate immune mechanism of the body.

Researchers were in a debate regarding the cut-off levels of vitamin D serum levels (25(OH)D) in the body, and most experts in the area suggested that if a level falling below 20 ng/mL is deficient, 20–30 ng/mL is insufficient and the level above 30 ng/mL is sufficient [52]. However, this notion has developed only by considering calcium homeostasis and bone health, rather than the immunomodulatory effects of vitamin D. In this respect, we may require new vitamin D threshold levels, which consider both bone and immunity health. It is important to note that vitamin D deficiency (where serum 25-hydroxyvitamin D levels are <20 ng/mL) and insufficiency (25(OH)D between 20 and 29 ng/mL) [52,53] are widespread in the current generation, specifically for people living away from the equatorial region [24,39,54]. This deficiency is also commonly observed in hospitalised patients suffering from various infections and is particularly relevant to those with ARTI and ARDS [10,11,44,55]. Most respiratory tract infections are spread during winter, strengthening the hypothesis that vitamin D deficiency is the main factor in lowering immunity in the absence of sunlight. Interestingly, the same hypothesis supported the relationship between vitamin D and rickets in 1897, when Kassowitz identified that the incidence of rickets’ was markedly increased during the winter season and decreased during the summer [56]. Even though it is noted that vitamin D deficiency may increase the risk of microbial infections, adequate supplementation will reduce the risk significantly against many infections, including dengue viral infection [57].

## 5. The Influence of Vitamin D in Reducing ARTI and ARDS

Apart from the effects of COVID-19, general respiratory tract infections occur worldwide, causing millions of fatalities annually (2.8 million in 2010) [58]. There have been several articles published, which have shown the relationship between vitamin D levels in the body and the potential occurrence of ARTI and ARDS [10,31,59,60,61,62].

**Figure 2:**
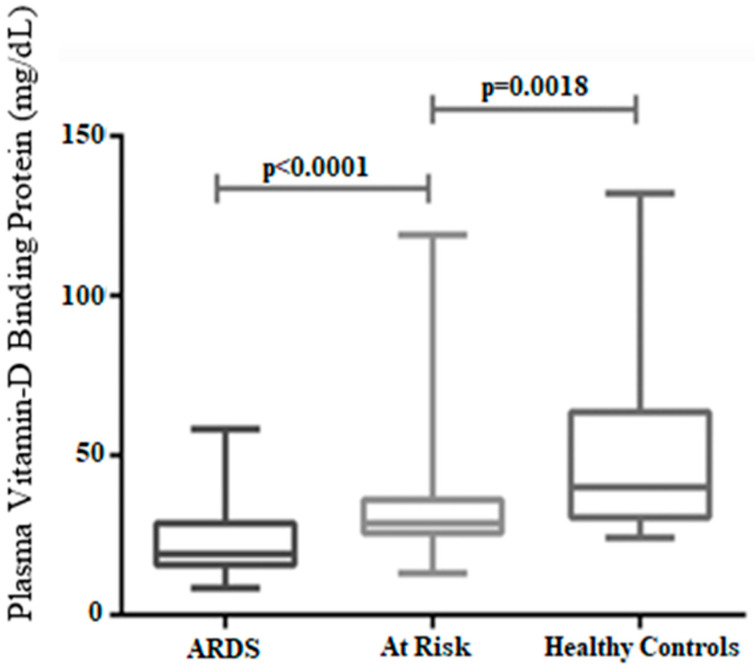
The association between plasma 25(OH)D_3_ levels and ARDS. The group with low 25(OH)D_3_ has ARDS, the moderate level is at risk, and those who have high vitamin D levels are healthy controls (adapted from [10]).

**Figure 3:**
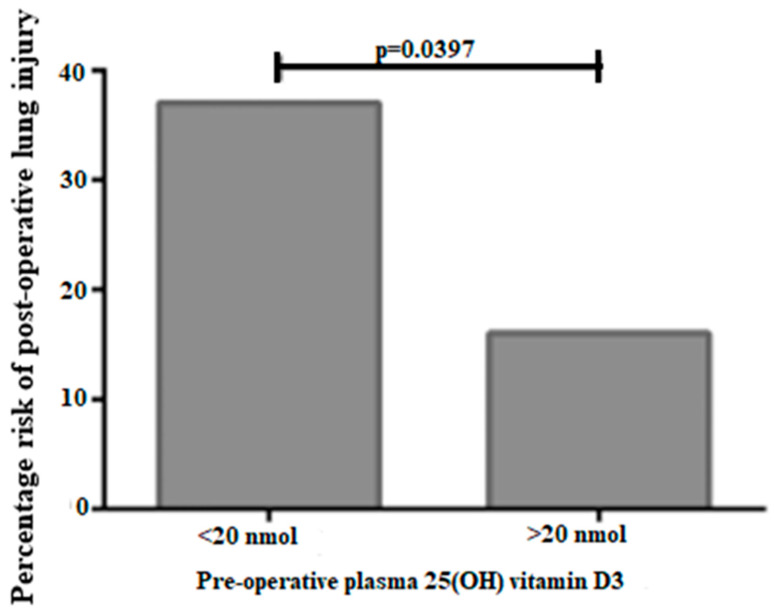
Relationship between severe 25(OH)D_3_ deficiency and risk of ARDS in the post-esophagectomy environment (adapted from [10]).

In an earlier study, Hansdottir and Monick [63] revealed that vitamin D levels are associated with the mitigation of viral respiratory tract infections and acute lung injury, which suggest that vitamin D is efficacious in providing protection against acute lung injury by modulating the expression of the renin–angiotensin system [19]. A randomized placebo-controlled trial demonstrated that vitamin D deficiency is a risk factor in developing acute lung injury [62], whilst a second lung function study demonstrated that vitamin D deficiency has a strong correlation with more rapid lung function decline [57]. In support of this link, ARDS development in a cohort of post-esophagectomy patients was found to be associated with a deficiency of vitamin D (Figure 2 and Figure 3) [10]. A murine model study also found that dietary-induced vitamin D deficiency contributes to exaggerated alveolar inflammation, epithelial damage, and hypoxia [10]. Similar results have emerged from researchers who reported that many esophagectomy patients suffer from ARTI and ARDS in the post-operative environment, and vitamin D supplementation during pre-operative days appears to reduce the risk of ARTI and ARDS in those cohorts [60]. They also found that vitamin D supplementation increased the concentration of 25-hydroxyvitamin D and 1,25 dihydroxy vitamin D in affected patients, which contributed to reduced lung inflammation [60]. Several studies have also showed a link between the vitamin D level and influenza infection [32,64,65,66].

A large meta-analysis of 25 eligible randomised controlled trials (11,321 participants, aged 0 to 95 years) demonstrated that vitamin D supplementation reduced the risk of ARTI among all participants by around 11% [11] and found that the protective effects were stronger (19%) in those who received daily or weekly supplementation. When the analysis took place in those groups, who had a vitamin D deficiency, the protective effects were highest for them, being around 70%. The overall conclusion was that vitamin D is a protectant against ARTI, and daily or weekly supplementation is better than a single bolus dose [11]. A similar large cross-sectional clinical trial has also shown the effects of lower vitamin D levels in increasing respiratory infection (*n* = 18,883) [67]. The deficiency effects were more pronounced in patients with underlying lung conditions [67]. Another meta-analysis of a randomised control trial was conducted in 2013, using data from 5660 patients in 11 placebo-controlled studies [68]. Patients with an average age of 16 years were provided with a vitamin D oral supplement of 1600 IU per day, with a daily dosing interval over three months. The results revealed that vitamin D supplementation is an effective method in limiting respiratory tract infections [68]. A fourth meta-analysis showed a significant correlation between an increase in vitamin D levels and a lower risk of respiratory tract infections [69]. In addition, a high dose of vitamin D administered to ventilated intensive care patients validated that it decreased the length of stay in the hospital environment [11,70].

Apart from the studies mentioned above, observational reports have shown a strong association between low ‘vitamin D (25(OH)D) levels and increased risk of respiratory tract infections (RTI). A large cross-sectional trial of more than 18,883 persons was conducted, and the results revealed an increase in RTI risk associated with lower 25(OH)vitamin D levels, which was significant in persons with chronic obstructive pulmonary disease (COPDs) [58]. Correlation between pre-hospital vitamin D status and the potentiality of incident acute respiratory failure in critically ill patients was studied, and a correlation was found between both lower vitamin D levels and a patient’s chance of infection [31]. Although vitamin D deficient patients have higher chances of mortality [31], it was found that timely vitamin D supplementation can mitigate this potentiality [70]. All these studies (Table 2) demonstrated that sufficient vitamin D levels in the body could prevent the occurrence of ARTI and ARDS in four ways: (i) elevating natural immunity, (ii) enhancing the antimicrobial response, (iii) inhibiting the production of pro-inflammatory cytokines, and (iv) improving the production of anti-inflammatory cytokines [41,71], which may act in COVID-19 patients.

## 6. The Link between Vitamin D levels, Geographical Context, Ethnicity, and Age

The coronavirus pandemic that spread in European countries and the United States in the initial stage (first wave) is claimed to have spread at a faster rate than in Asian and African countries during the same period [3]. This is unusual compared to the low population and population density in Europe and the US compared to south Asian countries. However, studies have shown that in a typical year, around 40% of people in Europe and those in the high latitudes of the US are vitamin D deficient [75,76]. This observation is consistent with the insufficient levels of ambient UV radiation required to synthesize a sufficient amount of vitamin D in high latitudes, coupled with cool summers and covered bodies for warmth [75,76].

### 6.1. Vitamin D and Geographical Context during the First Wave

It is interesting to note that the most initially affected COVID-19 regions such as the Wuhan area in China, Iran, Turkey, Italy, UK, France, Spain, and the USA are along the same latitudinal corridor with approximately similar climatic conditions (specifically low temperature and low humidity) and may be correlated with low serum vitamin D levels in the populations. The COVID-19 hospitalisation and mortality rates have also shown a similar latitudinal correlation with countries above 23.5° N latitude, specifically more fatalities per million during the first wave, except in Nordic countries. The average hospitalisation in the northern latitudes was 22%, and the mortality rate was 5.2% with respect to the confirmed cases, whereas the corresponding figures in the equatorial regions are much less (hospitalisation being 9.5% and mortality rate 3.1%) during the first wave [71]. The mortality rate, notwithstanding its definition as a percentage of confirmed cases or as the rate per million of population, is less in the equatorial region than the northern latitudes. The mortality rate with respect to the confirmed cases and deaths per million population in the equatorial or tropical regions until the end of August 2020 are 2.3% and 77, respectively, whereas the same figures in the northern latitudes (above 23.5° Lat) are 3.3% and 159, respectively. The mortality rate is also less in regions south of 23.5° S latitude (2.1%), but the deaths per million population are high (139) (Table 3). However, it is surmised that the gap between the northern latitude and equatorial regions will reduce shortly as the number of cases are increasing in the equatorial regions due to pressures related to higher population and population density.

The above figures are consistent with the high prevalence of solar radiation and vitamin D in the equatorial regions and its low prevalence in the northern latitudes. There are several observational studies already published, which emphasize the link between the vitamin D status of people in the northern latitudes (the worst-hit areas of COVID-19) and the occurrence of COVID-19 [7,27,67,77]. The serum vitamin D (25 (OH)D) status of 700 older Italian women (60 to 80 years old) was measured in the early part of the last decade, and it showed that 76% of these women fell into the deficient group [78]. It is suspected that if they were vitamin D deficient 17 years ago, it is likely that the situation might be continuing. This is based on the observation that more people may be sitting inside with electronic communication and entertainment devices, thus reducing their incidental solar exposure. Even though the equatorial regions have higher vitamin D compared to high latitude regions, some children in the former regions have been shown to have lower vitamin D levels, which has shown a correlation with asthma in these children [79].

Among the European countries, vitamin D status seems to be better in Nordic countries compared to Mediterranean countries. It is claimed that this better status is mainly because of the use of supplements, fortified foods, and more fatty fish and fish products in the diet [80]. This trend is generally coincident with COVID-19 fatalities, noting, however, that Sweden is an outlier.

### 6.2. Vitamin D and Ethnicity

Darker skin pigmentation and vitamin D synthesis demonstrate an inverse relationship [81]. This is consistent with the levels of fatalities in the US and UK, where black and brown-skinned people with high melanin levels in the skin have reduced amounts of vitamin D [24,80]. However, after analysing the data of 10,000 US children between the age of 1 to 21 years, researchers from National Health And Nutrition Examination Survey (NHANES) revealed that 9% (around 7.6 million) were vitamin D deficient (<10 ng/mL), and 61% (50.8 million) were vitamin D insufficient (between 10 and 20 ng/mL) [82]. This estimate does not appear to be consistent with the deficiency and insufficiency mentioned by earlier experts [52]. Nevertheless, if we consider these populations according to the vitamin D status mentioned by Holick [52], several additional millions fall in the deficient and insufficient category, removing the inconsistency. Other studies supported the claim that black and Hispanic ethnic groups have lower vitamin D serum levels compared to pale-skinned people in the US [83,84]. Of concern was that more black-skinned frontline health workers have died during this COVID-19 pandemic in the UK, and this may be because they may be very deficient in vitamin D. Vitamin D variation among various ethnic groups in Australia has also shown similar results, with deficiency seen in 80% of dark-skinned and veiled Australian women [53,85]. Of particular interest is that several studies have highlighted the influence of vitamin D in reducing ARTI and ARDS, as mentioned above, and this may have significant application in COVID-19 patients in reducing the severity and mortality.

### 6.3. Vitamin D and Old Age

It has commonly been reported that nearly half of the deaths occurring globally from the effects of COVID-19 are from aged care homes. Apart from a range of bodily infirmities, which naturally accompany old age, elderly people commonly have vitamin D deficiency, specifically due to aging systems, limited exposure to solar radiation, culture, and their living environment. This will include less renal and cutaneous synthesis and leads to a decrease in the level of 7-dehydrocholesterol in the skin [80,86,87,88]. After 65 years, there is a four-fold decrease in vitamin D production compared to a younger adult [39,54,89]. People of older age usually require more UVB exposure to synthesise sufficient vitamin D compared with the younger generation. It is found that in old age, osteoporosis is commonly associated with vitamin D deficiency due to the limited calcium absorption and hypersecretion of parathyroid hormone [90]. The presence of vitamin D receptors are also found in human muscles tissues, considered a nuclear receptor that binds with 1,25 hydroxyvitamin D [91]. Therefore, muscle weakness is an obvious symptom of vitamin D deficiency that may lead to falls and bone fractures, which can be rectified by vitamin D supplementation [91]. Vitamin D also has shown an association with cognitive performance in older adults with deficiency showing low mood, depression, and impaired cognitive performance [92]. A recent study from Ireland has been demonstrated that one in eight older adults are vitamin D deficient, which increases to one in five in winter, and further increases to one in two when examining the effect on the aging community (over 85 years) [93]. Reduction in vitamin D with age is also supported by Bilezekian et al. [41], who claimed that the vast majority of hospitalised elderly Italian patients presented with hypovitaminosis, with more than half exhibiting severe deficiency.

## 7. Vitamin D and COVID-19

The efficacy of vitamin D in resisting rhinovirus and secondary bacterial infection (upper respiratory tract infection) was also studied, and a positive response was found [94]. While there is little clear evidence linking sufficient vitamin D levels in the body with other diseases, it is still worth considering whether adequate vitamin D levels will mitigate similar respiratory virus symptoms, including COVID-19. There are some promising results from various parts of the world, including a number of observational studies, the details of which are mentioned in Table 4.

The influence of vitamin D as a predictor of poor prognosis for confirmed COVID-19 patients with acute respiratory failure was conducted by Carpagnano et al. [21] with a small cohort (*n* = 42) using a retrospective single-centre observational study. They analysed the vitamin D serum levels of 42 ARDS patients treated in a Respiratory Intermediate Care Unit (RICU) of the polyclinic in Bari (11 March to 30 April 2020) in Italy and found 81% of the patients had hypovitaminosis. Statistical analysis revealed that severe vitamin D deficient patients had a mortality probability of 50% after 10 days of hospitalisation, whereas it was reduced to 5% if the patient had a vitamin D level of >10 ng/mL [21]. Lau et al. [22] conducted a similar study using a cohort of 20 patients and found that 84.6% of patients admitted in the ICU were vitamin D deficient, whereas it was only 57.1% in the floor patients.

D’Avolio et al. [95] analysed the 25(OH)D level of 107 people tested for COVID-19 in a hospital in Switzerland and found that the median level of vitamin D was 22.2 ng/mL. This was similar to a control cohort in the same period in 2019, which showed a level of 24.6 ng/mL. However, with 27 people who tested positive with SARS-CoV-2, the median vitamin D (25(OH)D) level was significantly less, being only 11.1 ng/mL. With those who proved SARS-CoV-2 negative, the median vitamin D level was 24.6 ng/mL, and there was a significant difference between these levels (*p* < 0.004). A recently published study from Italy also showed that older people with Parkinson’s symptoms who took vitamin D supplements had only milder COVID-19 symptoms [96].

Tan et al. [97] conducted a small cohort observational study to evaluate the effect of vitamin D and vitamin B12, with magnesium (Mg) combination, on the severity of COVID-19 on older patients (>50 years). The study was conducted in a tertiary university hospital environment on 43 confirmed COVID-19 patients between 15 January and 15 April 2020. Out of the 43 patients, 17 received a combination of vitamin D, vitamin B12, and magnesium before the onset of the primary outcome, whilst the remaining 26 did not. In the multivariate analysis (after separately adjusting for age or hypertension), it has been observed that the interventional group retained a protective significance compared to the non-interventional group (17.6% vs. 61.5%, *p* = 0.006). In an effort to investigate the link between vitamin D concentration and COVID-19, Meltzer et al. [98] from the USA retrieved the one-year-old vitamin D status of 489 COVID-19 positive patients and conducted a comparative analysis. The results revealed that the risk of contracting COVID-19 by vitamin D deficient people is 1.7 times greater than those having sufficient vitamin D levels. Another observational study conducted in Germany with 185 COVID-19 positive patients also highlights a link between vitamin D deficiency and COVID-19 severity and mortality [99] Among the 185 patients, 41 (22%) were found to be vitamin D deficient, and they had a higher risk of ARDS, requiring mechanical ventilation, and an increased risk of death. A similar retrospective observational study, conducted in Israel with 7807 participants, included 782 COVID-19 patients. This study revealed a significant correlation between low serum vitamin D levels and the likelihood of COVID-19 [100]. In addition to this work, Baktash et al. [101] conducted a prospective cohort study in the UK among the older community (>65 years old). Their study also found that the COVID-19 positive group had a lower median serum 25(OH)D level (10.8 ng/mL) compared to the COVID-19 negative group (20.8 ng/mL).

It is also interesting to note the ability of vitamin D treatment to inhibit the SARS-CoV-2, which was shown in a study conducted in Singapore. Mok et al. [102] from the National University of Singapore have conducted excellent research to identify potential chemoprophylaxis against SARS-CoV-2 by performing virus-induced cytopathic effects (CPE). Somewhat surprisingly, calcitriol was found to be effective against the virus (SARS-CoV-2) with a 0.69 log10 reduction, which occurred upon post-treatment on Vero E6 cells and human nasal epithelial cells (hNECs) [102].

The treatment efficacy of vitamin D against COVID-19 has been brought to light by a university hospital in Spain [28]. In the Reina Sofia University Hospital, a group of researchers conducted a randomised open-label, double-masked clinical pilot trial on 76 patients. Although this was a small trial, the results were remarkable. The researchers divided the 75 COVID-19 positive patients (by RT-PCR test and radiographic pattern of viral pneumonia) into two groups (25 patients in group A and 50 patients in group B) randomly, and both groups received the standard care using the best available therapy. Apart from this standard care, group B patients were treated with calcifediol (25(OH)D) at a rate of 0.532 mg on day 1, followed by 0.266 mg on days 3 and 7, and then weekly until discharge or ICU admission. The objective was to assess the efficacy of vitamin D on ICU admission and mortality. The results showed that with group B (treated with calcifediol), only one patient (2%) required admission to the ICU. By comparison, those from group A (no treatment with calcifediol) had 13 patients (50%) who required admission to the ICU, followed by two deaths (Fischer test *p* < 0.001) [28]. Another significant highlight is that group B had 14 (28%) patients above 60 years of age, whereas in group A, it was 5 (19%) only. Even though this was a pilot study, it presages the efficacy of treating COVID-19 patients with 25(OH)D (calcifediol). Apart from this observation, a small cohort study (four patients) in the USA also found that those who received supplementation had lower oxygen requirements, an inflammatory marker reduction, and shorter hospital stay [103]. In support of this, Annweiler et al. [104] conducted a prospective cohort study in France and found that in the non-vitamin D arm, there was the occurrence of 55.5% mortality, whereas, in the intervention group, the mortality was only 17.5%. They concluded that bolus vitamin D supplementation during or just before COVID-19 was associated with less severity and a better survival rate in the elderly population [104].

**Table 4:** Some significant test results showing the link between vitamin D and COVID-19 (severity, mortality, and treatment).

Study Details/Objective(S)	Results/Conclusions	Reference(s)
Measured serum vitamin D levels of 42 COVID-19 patients with ARDS in a hospital in Italy.	It was found that the potential mortality of hypovitaminosis patients was 50% after 10 days of hospitalisation, whereas it was 5% for only those with vitamin D levels above 10 ng/mL	[21]
The study analysed the serum vitamin D levels of COVID-19 patients in a single hospital (small cohort).	The serum 25(OH)D levels of 20 confirmed COVID-19 patients found that 65% required ICU admissions. Among the ICU patients, 84.6% were vitamin D deficient than floor patients (57.1% only). Apart from this, 100% of the ICU patients less than 75 years of age were vitamin D deficient. Even though it was a small observational study, it highlighted a link between vitamin D deficiency and COVID-19 risk.	[22]
Probed the efficacy of vitamin D (calcifediol) in treating COVID-19 patients—specifically the link between ICU admission and mortality.	Seventy-five COVID-19 positive patients were randomly selected and divided into two groups: both received standard care, and one group received vitamin D (calcifediol) oral supplement as additional care. From the vitamin D treated group, only 2% went to ICU, whereas it was 50% from the untreated group. Treatment with vitamin D (calcifediol) significantly reduced the ICU admission and mortality.	[28]
A retrospective analysis in Switzerland investigated the association between vitamin D and COVID-19.	In this study, significantly lower serum 25(OH)D level obtained in COVID-19 positive (27 patients) (11.1 ng/mL) cohort compared to the negative patients (80 people) (22.2 ng/mL), which was comparable to that of the control group (*n* = 1377).	[95]
Influence of vitamin D/ B12 and Mg combination was investigated in older (above 50 years) COVID-19 patients.	The result has shown that the combination therapy reduced the need for oxygen therapy and/or ICU support.	[97]
Investigated the link between vitamin D and COVID-19 severity, including mortality.	The study revealed a link between severe vitamin D deficiency and COVID-19 severity and mortality.	[99]
Researchers compared the COVID-19 test results of 14,000 people with their previous vitamin D levels.	The mean vitamin D level was significantly lower in the cohort found positive for COVID-19, compared to those who tested negative. Low plasma 25(OH)D concentration was found to be an independent risk factor for COVID-19.	[100]
This study assessed the significance of vitamin D in older COVID-19 patients.	The study found that the COVID-19 positive group had a lower median serum 25(OH)D level (10.8 ng/mL) compared to the negative group (20.8 ng/mL).	[101]
In the absence of vaccines and proper treatment, probed the potentiality of chemoprophylaxis.	An in vitro study has shown that calcitriol (vitamin D) among various potential libraries was found to be effective against SARS-CoV-2 with 0.69 log_10_ inhibition in human nasal epithelial cells (in vitro). If the result is replicated in clinical trials, host-directed therapy receives consideration, and calcitriol can be used as ring prophylaxis of the contacts of COVID-19 patients.	[102]
Researchers have given vitamin D oral supplementation to four COVID-19 confirmed patients—cholecalciferol 1000 or ergocalciferol 50,000 IU/day/5 days.	Patients who received high dose supplementation achieved normal vitamin D levels, which was seen in their clinical recovery level (lower oxygen requirements, reduction in inflammatory marker status, and shorter length of stay).	[103]
Determined the efficacy of bolus vitamin D3 supplementation during or just before COVID-19 in elderly adults.	They observed that 82.5% of the intervention group survived COVID-19, whereas only 44.4% survived in the comparator group.	[104]
The vitamin D serum levels of two cohorts of COVID-19 patients (active and expired) were statistically analysed.	Univariate analysis results showed that vitamin D deficiency is associated with the odds of death. While controlling the age, sex, and comorbidity, vitamin D deficiency has shown a strong association with COVID-19 mortality.	[105]

## 8. The Role of Vitamin D in Infection Control

Most of the patients with respiratory diseases, including COVID-19, were found to be associated with vitamin D deficiency, implying that vitamin D supplementation or solar radiation exposure might improve their potential for healing [106]. Studies pointed out that vitamin D influences both the innate and adaptive arms of immunity, particularly in making proper cell signalling pathways due to the presence of vitamin D receptors (VDR) in the immune cells [107,108]. In summary, vitamin D may:

- Reduce microbial (including viral) respiratory tract infections [74,107,109,110];
- Boost immunity through the induction of antimicrobial peptides such as cathelicidins (hCAP-18/LL-37) and defensins, which can lower the viral replications in the body [44,46,110,111];
- Reduce cytokine and chemokine storms, and reduce the production of pro-inflammatory cytokines, tumour necrosis factor-ἁ, and interferon-γ—which usually produce inflammation in lung alveoli causing ARDS [46,74,109,110,111,112];
- Increase the expression of anti-inflammatory cytokines [110];
- Promote the production of regulatory T cells, which inhibit the inflammatory process [113];
- Act as a negative regulator of RAS in the form of 1,25 dihydroxyvitamin D/VDR [114,115];
- Maintain pulmonary epithelial barrier integrity and stimulate epithelial repair [43,116]; and
- Protect against ALI through calcitriol/VDR signalling [17].

Angiotensin-Converting Enzyme-2 (ACE-2) receptors are highly expressed on type II pneumocytes; therefore, they are primary targets for SARS-CoV-2. Bombardini and Picano [117] are of the opinion that impaired functions of these cells decrease their surfactant levels and thus increase their surface tension, making it more favourable for the SARS-CoV-2 to infect the cells. However, it was reported that 1,25 dihydroxyvitamin D can stimulate surfactant levels in alveolar type II cells during in vitro studies [118,119], which potentially makes the situation detrimental for the SARS-CoV-2 to infect the ACE-2.

In line with the current COVID-19 seriousness and the need for treatment methodology, various previous clinical studies have shown that treatment with high doses of vitamin D (250,000–500,000 IU) is safe for both critically ill and mechanically ventilated patients [70,120]. This treatment has helped to increase the haemoglobin level and oxygen-carrying capacity of blood in patients and reduce their length of stay in the hospital environment [70,120]. In another study by Sabetta et al. [64], it was found that acute viral respiratory tract infection was two-fold less prevalent in patients if their vitamin D serum levels were above 36 ng/mL (hazard ratio 0.51, 95% CI, 0.25–0.84, *p* < 0.0001) and the percentage of sick days was five times less in comparison with other patients whose vitamin D serum levels were less than 38 ng/mL [64]. In an in vitro study, vitamin D also showed antiviral activity against rhinovirus in human epithelial cells [121]. It thus seems likely that maintaining sufficient levels of vitamin D in the serum is a significant issue in vulnerable people in the community, such as older adults, those that are immunocompromised, and patients with chronic conditions. In these situations, such people’s bodies are already pre-set for an elevated level of inflammatory response if exposed to SARS-CoV-2 infection, as mentioned by Laird et al. [7].

## 9. Acquiring Sufficient Levels of Vitamin D

Exposure to sunlight has traditionally been considered a therapy for several illnesses [122], but as indicated earlier, there is a progressive fall in UVB radiation and subsequent vitamin D production with a progressive increase in distance from the equator [24,123]. Exacerbating the issue is that there has been recent advice to limit sun exposure because of the correlation between high UV exposure levels and the increased risk of skin cancer (cell carcinoma and melanoma) [124]. In ideal conditions, around 50% to 90% of the required vitamin D in the body develops on the skin from UVB radiation, with the remainder having to be supplied through diet [24,82]. However, notwithstanding the specific barriers introduced by modern living conditions, there are suitable methods to acquire enough vitamin D levels, including:

1. Exposing the body sensibly to natural solar radiation;
2. Consuming a vitamin D-rich diet consisting of: egg yolk, cod liver oil, vitamin D fortified dairy products/juice/or other foods, wild mushroom, oily fish such as tuna, mackerel, herring, sardine, and wild salmon;
3. Regularly taking vitamin D supplements in tablet form (recommended under medical supervision to those who have any health risk).

Although exposing the body to solar radiation is the best and the cheapest option to develop sufficient vitamin D, revealing enough skin to ambient sunlight and the calculation of optimal exposure is a complicated procedure, as there are several parameters involved, including climate, weather, location (latitude and elevation from the mean sea level), atmospheric pollution, and area of the body exposed [24,123,125,126]. Serrano et al. [126] analysed the solar ultraviolet erythemal (UVER) irradiance (W/m^2^) at Valencia in Spain from 2003 to 2010 to estimate the solar UV radiation and the exposure time necessary for the production of the recommended daily dosage of vitamin D (1000 IU) and found that it varies from 7 minutes in summer to around 2 hours in winter. For exposure between 10 a.m. and 3 p.m., where there are high UVB radiation levels, the optimum time and duration of solar exposure should be advised by the local meteorological and/or health authorities. The second method of obtaining vitamin D is from consuming vitamin D-rich food, but it may prove challenging to receive sufficient vitamin D from food alone [58]. The third route is the regular intake of supplements, which is useful for people who have low sun exposure and dietary restrictions. According to the US National Academy of Medicine, an adult requires around 600–800 IU of vitamin D per diem, with no variation being noted between men and women. In 2016, the scientific panel on Dietetic Products, Nutrition and Allergies (NDA) of the European Food Safety Authority (EFSA), defined adequate intakes (AIs) of vitamin D for all population groups as shown in Table 5. This general recommendation was made without taking any infection control or pandemic issues into consideration. These previous studies showed that vitamin D supplementation should be beneficial to those with deficiency, and further, that daily or weekly supplementation is more beneficial compared to the bolus dose [11]. However, a randomized controlled double-masked pilot study conducted in Spain has shown that the bolus dose is also effective in treating COVID-19 [28]. Though large-scale controlled study results have not yet been published, it appears better to recommend a high dose of vitamin D to the COVID-19 patients and people under quarantine as adjuvant therapy in support of other recommendations [127]. In such cases, calcifediol’s direct supplementation is highly recommended as it can quickly raise the serum vitamin D level [28]. Though the recommended dosage of vitamin D supplementation is normally safe, prolonged use of very excessive supplementation may create hypervitaminosis D leading to hypercalcemia, with subsequent renal and cardiovascular damage [128]. By taking this excess effect into account, the Food and Nutrition Board of the Institute of Medicine of the National Academies in the US has recommended 4000 IU per day as the upper recommended level for adults [129]. They further suggested that the circulating vitamin D (25(OH)D) level above 50–60 ng/mL (125–150 nmol/L) should not be allowed as it can create cardiovascular and renal complications [129]. Even though several vitamin D oral supplementation recommendations have been proposed, specifically in recent times due to the COVID-19 pandemic, it appears that it will be more effective and healthier if the recommending agencies consider the following factors: (i) initial vitamin D level; (ii) living environment (latitude, types of work); (iii) skin pigmentation; and (iv) obesity.

**Figure 4:**
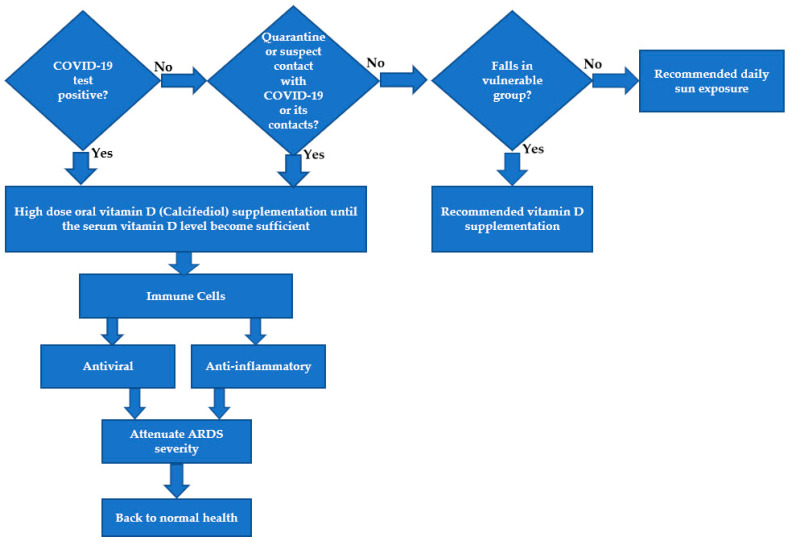
Suggested vitamin D pathways for COVID-19 patients, the vulnerable group, and the general public.

Based on several observational studies [21,22,97,99,102,105] and through a randomized pilot study conducted in Spain [28], it was found that people with COVID-19 require a sufficient level of vitamin D to prevent severity. The effects of vitamin D will be more significant in people with low vitamin D levels. Therefore, we suggest that a high dose of oral calcifediol supplementation be offered to COVID-19 patients immediately after the confirmation of their status and also to people under quarantine conditions (Figure 4). Supplementation is also suggested for vulnerable people in the community as either calcifediol or cholecalciferol, and other people can be vitamin D sufficient through adequate solar exposure. The later mode is clearly the cheapest option with the caveat of considering the potential for melanoma in pale-skinned people.

## 10. Conclusions

Vitamin D is unarguably a significant factor in general bodily health, but of particular interest in the context of COVID-19, it is correlated to a positive impact in reducing infection and symptoms. Hypovitaminosis of vitamin D results in many conditions such as rickets, osteoporosis, osteomalacia, certain cancers, hypertension, cardiovascular disease, and can be considered a significant factor in the development of cytokine storms that lead to ARDS among COVID-19 patients. At this time (December 2020), no large, high-quality randomised placebo-controlled multicentral study has been published that establishes vitamin D level measurements or high dose vitamin D performance on COVID-19 patients. However, the results of existing observational, small cohort, and pilot studies are promising. Therefore, it is suggested that measurements of vitamin D levels in all COVID-19 confirmed patients in various stages of the diseases would lead to a significant database that would assist in understanding the relationship between vitamin D levels and significant COVID-19 symptoms, which will allow the creation of an evidential model. Work to date also suggests that increasing vitamin D levels in all COVID-19 patients will alleviate the severity of symptoms and mortality.

Vitamin D serum level deficiency is commonly seen in older adults, those with obesity, those having pre-existing chronic conditions, and people living in high latitudes, specifically with darker skin pigmentation. This is considered to be the vulnerable group in respect to COVID-19, which is based on the correlation of low vitamin D levels and COVID-19 seriousness and mortality. Whilst there are various other medical explanations, the data lead to the suggestion that solar exposure and raised vitamin D serum levels can alleviate the severity of COVID-19 symptoms. Information from all the previous studies suggests that a sufficient level of vitamin D serum level in the body can move a person from pro-inflammatory levels to anti-inflammatory levels, which can save the lives of the majority of COVID-19 patients. Therefore, an adequate level of exposure to solar radiation or supplementation of vitamin D should be considered as prophylactic against COVID-19, which may be very beneficial to low- and middle-income countries. Notwithstanding this evidence, additional evidence from well-designed placebo-controlled studies is required to more clearly understand the therapeutic potential of vitamin D regarding COVID-19 and many other similar diseases. However, waiting for the results of a comprehensive study for several months (without suggesting vitamin D) cannot be tolerated as tens of thousands of people are succumbing to the virus each day.

## Figures and Tables

**Table 1:** Clinical symptoms similar to COVID-19 and its relationship with vitamin D (from previous studies).

Symptoms/Characteristics	Relation to Vitamin D	References
Acute respiratory distress syndrome (ARDS) risk	Inverse correlation	[10,31]
Severe pneumonia	Inverse correlation	[20,32,33]
Sepsis risk	inverse correlation	[34,35]
Pro-inflammatory	Inverse correlation cytokine production	[36,37]
C-Reactive Protein increase	Inverse correlation	[38]

**Table 2:** Selected previous studies show the association between vitamin D and ARDS, including lung injury.

Study Details	Results/Conclusion	Reference(s)
Determined the relationship between vitamin D and ARDS.	Vitamin D deficiency was ubiquitous with ARDS patients and was a risk factor in developing ARDS following the esophagectomy. It also found that the pharmacological repletion of vitamin D before esophagectomy reduced the alveolar–capillary damage compared to the vitamin D deficient patient. In a murine model, dietary-induced vitamin D deficiency generated alveolar inflammation, damage in epithelial cells, and hypoxemia.	[10]
Investigated the role of supplemented vitamin D in reducing ARTI in a meta-analysis. This study used 25 randomised controlled trial data from to 11,321 participants.	The study found that (i) vitamin D supplementation reduced the risk of ARTI in all patients (odds ratio-OR-0.88); (ii) protective effects were higher in those receiving a daily or weekly dose compared to the bolus dose (OR-0.81); and (iii) among the second group, protective effects were more potent in those who had vitamin D deficiency (<10 ng/mL). The overall conclusion is that vitamin D supplementation protected from ARTI.	[11]
Investigated whether vitamin D alleviates lipopolysaccharide (LPS)-induced acute lung injury (ALI).	Vitamin D exhibited positive effects to LPS-induced ALI in rats by modulating the expression of members of the renin–angiotensin system (RAS) such as angiotensin (Ang-1) converting enzyme (ACE and ACE2), renin and Ang-II.	[19]
Investigated the association between pre-hospital vitamin D level and acute respiratory failure.	The study found an association between pre-hospital vitamin D levels and the risk of incident acute respiratory failure in critically ill patients, including death.	[31]
Investigated the role of vitamin D supplementation in patients undergoing esophagectomy.	Vitamin D supplementation 3–14 days prior to the esophagectomy significantly reduced the potentiality of ARDS during the post-operative environment.	[60]
Investigated the association between vitamin D and ALI and ARDS through a review.	Reviewers found a profound role of vitamin D in modulating the immune response and a potential role in ALI.	[62]
Investigated the association between vitamin D and acute respiratory infection (ARI) through systematic review.	The review identified a consistent association between vitamin D deficiency and risk to ARI.	[68]
Vitamin D deficiency and ARDS.	Vitamin D deficiency was found to be prevalent (90%) among 476 ventilated patients with ARDS, also associated with a longer duration on mechanical ventilation	[72]
Investigated whether exogenous vitamin D attenuates lipopolysaccharide (LPS)-induced lung injury via modulating the epithelial cell proliferation.	It was found that vitamin D supplementation attenuates lung injury via (i) stimulating alveolar epithelial type II (ATII) cell proliferation and migration; (ii) reducing epithelial cell apoptosis; and (iii) inhibiting the transforming growth factor (TGF-β) induced epithelial–mesenchymal transition (EMT). The study suggested that vitamin D has therapeutic potential for the resolution of ARDS.	[73]
Investigated whether the vitamin D or VDR pathway ameliorates LPS-induced ALI (mice model).	It was found that vitamin D treatment alleviated the LPS-induced lung injury.	[74]

**Table 3:** Total confirmed cases, total mortality, mortality rate with respect to the confirmed cases, confirmed cases per million, and deaths per million of the population (of COVID-19) as of 10 September 2020. Note the low mortality rate and the number of deaths per million in the equatorial region compared to northern latitudes (above 23.5° N).

Location	Total Confirmed Cases	Total Mortality	Percentage of Mortality to Confirmed Cases	Confirmed Cases Per Million Population	Deaths Per Million Population	Total Population
North of 23.5° N Lat	13,201,342	483,004	3.3	5378	159	3,655,796,736
23.5° N to 23.5° S	12,320,973	364,084	2.3	3295	77	3,918,475,190
South of 23.5° S Lat	1,589,874	37,423	2.1	5811	139	197,607,413

**Table 5:** The adequate intake (AI) of vitamin-D for all populations as recommended by the European Food Safety Authority (EFSA) [130] and the American National Academy of Medicine (ANAM) [129].

Age	EFSA (mcg/day)	(IU/day)	ANAM (mcg/day)/(IU/day)
1–11 months	10	400	-
1–18 years	15	600	-
>18 years	15	600	20/800

Note: mcg—microgram; IU—international unit. For persons with existing health problems, especially cardiac and renal issues, a local doctor should be consulted before taking any supplement.

## Data Availability

Not applicable.
